# Cochlear Implant and Its Related Science

**DOI:** 10.1155/2015/683967

**Published:** 2015-07-09

**Authors:** Chung-Feng Hwang, Yang Chen, Hung-Ching Lin, Prepageran Narayanan, Seung-Ha Oh, Eric Truy

**Affiliations:** ^1^Department of Otolaryngology, Kaohsiung Chang Gung Memorial Hospital and Chang Gung University College of Medicine, Kaohsiung 83301, Taiwan; ^2^Department of Speech-Language Pathology, Duquesne University, Pittsburgh, PA 15282, USA; ^3^Department of Audiology and Speech Language Pathology, Mackay Medical College, Taipei 10449, Taiwan; ^4^Department of Otorhinolaryngology, Head and Neck Surgery, University of Malaya, 506030 Kuala Lumpur, Malaysia; ^5^Department of Otolaryngology, Seoul National University College of Medicine, Seoul 110-744, Republic of Korea; ^6^ENT Departments, Hôpital Femme-Mère-Enfant and Edouard Herriot University Hospitals, Claude Bernard University, 69003 Lyon, France

The prevalence of hearing loss (presbycusis) is 35–50% in those aged 65 years or older; consequently, hearing assistant devices become more and more important [1]. Cochlear implantation (CI) is believed to be one of the most important technologic achievements to have occurred in the 20th century for the treatment of profound hearing loss, continually improved since its approval by the International Consensus Conference in 1995 [2]. Recent advances in biology and medicine have introduced new concepts in the study of CI and its related science. Many changes have taken place including improvements in hardware technique, expansion of candidacy, and clinical outcome. The cornucopia of all novel technologies and approaches serves as important blessings for hearing-impaired people. This special issue is to exhibit the diversity and advances in recent progress that contributes to the different subspecialties of CI and its related science.

It has motivated intense investigation on developing stem cell therapy as a new therapeutic strategy, for example, through the transplantation of stem cells into the inner ear for hearing restoration [3]. H.-C. Chen et al. investigated the long-term effect of hypoxia on stemness and the bioenergetic status of cochlear stem/progenitor cells cultured at different low oxygen tensions.

Recent advances in hearing preservation studies have introduced new concepts and technologies to be applied in CI [4, 5]. To develop skills sufficient for hearing preservation CI surgery, surgeons need to perform several electrode insertion trials in ex vivo temporal bones, thereby consuming relatively expensive electrode carriers. J.-P. Kobler et al. design low-cost dummy electrodes that are cheap alternatives for surgical training and for in vitro, ex vivo, and in vivo research purposes. P. T. Bhatti et al. also present an effective method for tailoring the flexibility of a commercial thin-film polymer electrode array for intracochlear electrical stimulation.

The benefits of residual hearing preservation in cochlear implant recipients have promoted the development of atraumatic surgeries. The surgeons prefer round window approach to preserve low frequency hearing [6]. The incidence and severity of intracochlear trauma were not influenced by electrode array insertion through the anterosuperior or anteroinferior quadrant of the round window membrane.

A bone-anchored hearing aid (BAHA) or bone-anchored hearing device is a type of hearing aid based on bone conduction [7, 8]. They are more expensive than conventional hearing aids, and their placement involves invasive surgery which carries a risk of complications [8]. The use of a wide fixture implant and the nonskin thinning surgical technique indicates that the combination is a safe procedure with good stability and no abutment losses in M. Hultcrantz's research.

The diagnostic value of high resolution computed tomography (HRCT) and magnetic resonance imaging (MRI) before CI is very high [9]. M. Busi et al. suggest that CI is a safe and effective procedure even for patients with brain and inner ear abnormalities at neuroimaging investigations with HRCT and MRI. Nonetheless, common cavity and stenosis of the internal auditory canal (less than 2 mm) are negative prognostic factors even if brain lesions are absent.

Limiting the assessment of CI performance strictly to speech perception improvement does not properly evaluate the characteristics of the prosthesis-neural interface. Electrophysiological testing should provide a more accurate proxy of the interaction between the electrodes of the CI and the auditory neurons. F. Venail et al. modeled the activation of auditory neurons in CI recipients. Distribution of Neural Responses Telemetry residues could provide a proxy of auditory neurons functioning in implanted cochleas.

The outcome of CI varies over a wide range among pediatric patients [10]. M. Park et al. assess the correlation between performance intelligence and postoperative CI outcome. Performance intelligence, especially social cognition, was strongly related to the postoperative CI outcome. Therefore, auditory rehabilitation, including social rehabilitation, should maximize the postoperative CI outcomes.

According to H.-S. Hsieh et al., implanted children tend to write stories that are shorter, worse organized, and without a plot, while formulating morphosyntactically correct sentences. Special attention is required on their auditory and language performances, which could be the underlying causes of the written language problems.

In this special issue, we collected both basic and clinical original research articles stimulating the continuing efforts to understand the cochlear implant technology, the development of strategies to treat deafness, and the evaluation of outcomes. It is our wish to increase interest in CI and its related science research with this special issue and further accelerate the development of novel therapies for hearing impairment.

## References

[1] Parham K., McKinnon B. J., Eibling D., Gates G. A. (2011). Challenges and opportunities in presbycusis. *Otolaryngology—Head and Neck Surgery*.

[2] Jaekel K., Richter B., Laszig R. (2002). The history of cochlear implantation: from Volta to multichannel-intracochlear stimulation. *Laryngo-Rhino-Otologie*.

[3] Nishimura K., Nakagawa T., Ono K. (2009). Transplantation of mouse induced pluripotent stem cells into the cochlea. *NeuroReport*.

[4] Gstoettner W., Kiefer J., Baumgartner W.-D., Pok S., Peters S., Adunka O. (2004). Hearing preservation in cochlear implantation for electric acoustic stimulation. *Acta Oto-Laryngologica*.

[5] Miranda P. C., Sampaio A. L., Lopes R. A., Ramos Venosa A., Oliveira C. A. (2014). Hearing preservation in cochlear implant surgery. *International Journal of Otolaryngology*.

[6] Skarzynski H., Lorens A., Piotrowska A., Anderson I. (2007). Preservation of low frequency hearing in partial deafness cochlear implantation (PDCI) using the round window surgical approach. *Acta Oto-Laryngologica*.

[7] Janssen R. M., Hong P., Chadha N. K. (2012). Bilateral bone-anchored hearing aids for bilateral permanent conductive hearing loss: a systematic review. *Otolaryngology—Head and Neck Surgery*.

[8] Banga R., Lawrence R., Reid A., McDermott A.-L. (2011). Bone-anchored hearing aids versus conventional hearing aids. *Advances in Oto-Rhino-Laryngology*.

[9] Bettman R., Beek E., Van Olphen A., Zonneveld F., Huizing E. (2004). MRI versus CT in assessment of cochlear patency in cochlear implant candidates. *Acta Oto-Laryngologica*.

[10] Hwang C.-F., Chen H.-C., Yang C.-H., Peng J.-P., Weng C.-H. (2012). Comparison of Mandarin tone and speech perception between advanced combination encoder and continuous interleaved sampling speech-processing strategies in children. *American Journal of Otolaryngology—Head and Neck Medicine and Surgery*.

